# Lipid content and fatty acid composition of *Porosira glacialis* and *Attheya longicornis* in response to carbon dioxide (CO_2_) aeration

**DOI:** 10.1371/journal.pone.0177703

**Published:** 2017-05-18

**Authors:** E. Y. Artamonova, T. Vasskog, H. C. Eilertsen

**Affiliations:** 1 Department of Arctic and Marine Biology, UiT The Arctic University of Norway, Tromsø, Norway; 2 Norut Northern Research Institute, Tromsø, Norway; The Education University of Hong Kong, HONG KONG

## Abstract

In the current study two novel psychrophilic diatoms *Porosira glacialis* and *Attheya longicornis* were tested for suitability to CO_2_ mitigation coupled with production of the physiologically requisite omega– 3 fatty acids. This study is in line with the worldwide conducted research aimed at applying *biorefinery* concept to heavy polluting industries. Since the production of algal high value compounds, i.e. essential fatty acids, relies on utilization of residual CO_2_ emissions coming from industry, the costs of such production maybe substantially reduced. Besides, the ecological benefits of the biorefinery concept being implemented are obvious, since CO_2_ is one of the major greenhouse gases. The current research has shown that one of the tested microalgal species, the diatom *P*. *glacialis* showed good tolerance to high (20–25%) levels of CO_2_ and maintained growth rates comparable to controls. The total lipid content in the CO_2_ aerated culture increased from 8.91 to 10.57% in cell dry mass. Additionally, the content of docosahexaenoic acid (DHA) increased from 3.90 to 5.75%, while the concentration of eicosapentaenoic acid (EPA) decreased from 26.59 to 23.66%. In contrast, *A*. *longicornis* did not demonstrate any significant increase in total lipid content. Besides, its growth was hampered by high levels of CO_2_ aeration.

## Introduction

Nowadays mitigation of extensive CO_2_ emissions is considered urgent due to the anthropogenic factor, i.e. outlet from industry and other human activities. Plant flue gases containing CO_2_ are one of the main concerns, since industrial contribution to the total world CO_2_ emissions is more than 7% [1, 2]. Several solutions to reduce CO_2_ pollution have been suggested, and biological sequestration by microalgae is a “hot” candidate here since microalgal photosynthetic efficiency and growth rates by far surpasses those of terrestrial plants [3]. Besides that, microalgae are considered to be promising candidates for production of biodiesel, high prized food/feed supplements, cosmetics, etc. [4, 5]. A recent review by Koller et al. (2014) [6] reported market prices for algae-derived EPA and DHA to be as high as 4600 and 50 US $ per kg accordingly. The assessed global market volumes for DHA is impressive 4 × 10^8^ US $ (estimated for the USA). On the other hand, the evaluated costs of CO_2_ removal from a power plant is US $ 128–967 per ton of carbon, that is by far surpasses the economically feasible: US $ 10 per ton of carbon [7].

Industrial mass cultivation of microalgae at high densities requires addition of CO_2_ well above the levels available in seawater and/or by common aeration. Thus, Li et al (2013) [8] demonstrated that for each ton of microalgal biomass production, at least 1.83 t of CO_2_ is required.

There are quite a number of reports on how CO_2_ aeration influences the growth and lipogenesis of microalgae. Some studies concluded that CO_2_ aeration above 5% may be harmful for microalgae, e.g. Chang and Yang (2003) and Silva and Pirt (1984) [2, 9], while others showed that CO_2_ supply as high as 10% [10, 11] or even more [12] did not have any negative effects on the microalgal growth. Further, CO_2_ aeration was shown to be beneficial in terms of growth, total lipid content and the rate of fatty acid unsaturation in a number of studies [3, 10, 11, 13]. Thus, it was demonstrated that the lipid productivity of *Scenedesmus* sp. and *Botryococcus braunii* significantly increased when aerated with flue gas containing 5.5% CO_2_ [10]. Chiu et al. (2009) [3] found that *Nannochloropsis oculata* had maximum growth and lipid productivity when aerated with air containing 2% CO_2_. *Chlorella pyrenoidosa* and *Scenedesmus obliquus* demonstrated best growth potential at 10% CO_2_, while higher CO_2_ levels (30–50%) triggered accumulation of total lipids and polyunsaturated fatty acids [10]. Similarly, Watanabe et al. (1992) [11] showed that 10% CO_2_ was optimal for growth of *Chlorella* sp. Nakanishi et al. (2014) [14] demonstrated that CO_2_ supply (4%) triggered growth and lipid productivity of *Chlamydomonas* sp.

Mitigation of CO_2_ coming from thermoelectric plants or heavy polluting industries combined with e.g. production of high quality lipids (EPA, DHA etc.) may considerably decrease production costs of the algae biomass. This is why selection of appropriate microalgae species capable of efficient CO_2_ utilization and production is a main challenge today.

Our study was therefore aimed at investigating the potential of northern diatoms to satisfy the above mentioned criteria. Northern diatoms have, for natural reasons, low light—and temperature optima, this due to evolutionary adaptations. Additionally, beneficial fatty acid composition, with phylum—characteristic, elevated EPA content [15] and high growth rates [16] make northern diatoms promising candidates for mass cultivation.

## Material and methods

### Ethics statement

The present study on the cold water northern diatoms *Porosira glacialis* and *Atteya longicornis* was performed in accordance with the laws and did not investigate any endangered species. No specific permissions were required to collect phytoplankton samples in the investigated area of the Barents Sea and the coast of the northern Norway.

### Diatom cultivation with ambient air and air enriched with CO_2_

Monocultures of the large (50 μm) *Porosira glacialis* and the small (10 μm) *Atteya longicornis* diatom species originated from specimens isolated from spring bloom vegetative stocks collected in the Barents Sea (80°N, *P*. *glacialis*) and the coast of northern Norway (70°N, *A*. *longicornis*). For species identification, morphological and molecular methods were applied as described by Huseby [17]. The stock monocultures (5 mL) were initially inoculated in F/10 cultivation medium (35 mL) in 50 mL Nunclon sterile culture flasks [18]. Having reached sufficiently high densities, the cultures were transferred to sterilized 10 L plastic cultivation bottles kept at 5°C at scalar irradiance 33.2 μEs m^-2^ s^-1^ (measured with a Biospherical Instruments INC. QSL– 100) and photoperiod (Light: Dark) 14:10.

When the cultures became dense enough (> 1 000 000 cells/L), they were used to inoculate 100 L plexi columns with external LED illumination (“North light” LS 1768B-60, China) at the same irradiance and temperature conditions as for the inoculum. The diatoms were cultivated in a semi-continuous regime i.e. with partial replacement of medium when high densities were achieved.

*In vitro* chlorophyll *a* (Chl *a*) was measured three times each week with a Turner TD-700 fluorimeter as described by Holm—Hansen and Riemann (1978) [19] though using ethanol as extraction solvent. Growth rates (doublings per day) were calculated from the Chl *a* values as follows:

$$\mu =\frac{log2N_{1}-log2N_{2}}{d}$$

where *N*1 and *N*2 are Chl *a* at the beginning and at the end of the measurement period, and *d* is the number of days between *N*1 and *N*2.

The microalgae were harvested several times at high densities (see Table 1) in the exponential growth phase. Vacuum filtration (0.3 ATM) onto 5 μm (*A*. *longicornis*) or 20 μm (*P*. *glacialis*) pore size plankton mesh (Sefar Nytal R) was used for the biomass harvesting. The microalgal sludge was thereafter transferred to a beaker containing F/10 medium and centrifuged at 1314 g for 5 minutes at 4°C. Then the pellet was freeze dried overnight.

**Table 1:** The growth conditions of the microalgae.

Species	Temperature	Light intensity	Photoperiod	Cell density at harvest (*10^6^ cells L^-1^)
*A*. *longicornis* (controls)	5°C	33,2 μEs m^-2^ s ^-1^	14h:10h light: dark	52.8
*P*. *glacialis* (controls)	5°C	33,2 μEs m^-2^ s ^-1^	14h:10h light: dark	3.3
*A*. *longicornis* (CO2-treated)	5°C	33,2 μEs m^-2^ s ^-1^	14h:10h light: dark	136.3
*P*. *glacialis* (CO2-treated)	5°C	33,2 μEs m^-2^ s ^-1^	14h:10h light: dark	1.7

The cultures subjected to CO_2_ aeration had the same environmental conditions (temperature, inorganic nutrients, irradiance) as the controls (see Table 1). The supply of CO_2_ varying between 20 and 25% was provided to the microalgae culture during three days prior to the harvesting. For this purpose, ambient air aeration mixed with CO_2_ coming from a 40 L pressured (200 atm) CO_2_ steel tank (AGA, Oslo, Norway) was used. The harvesting procedures were the same as for the controls.

### Total lipid analysis

Total lipid analysis was performed as described in Cequier—Sanchez et al. (2008) [20] with some modifications. Total lipid from ca. 0.5 g for each triplicate of dry matter was extracted twice by immersion in 2 mL CH_2_Cl_2_/MeOH (2:1, v/v) and 2 mL 5% NaCl in glass vials. The samples were filtered with subsequent centrifugation at 1882 g at room temperature for 4 minutes. The organic liquid phase containing lipids was evaporated to dryness and the total lipids were estimated gravimetrically.

### Fatty acid composition analysis

Total fatty acid analysis was performed applying a modified method by Stoffel et al. (1959) [21]. The samples obtained from the total lipid analysis were resuspended in CH_2_Cl_2_/MeOH to a concentration of 10 mg per mL and frozen at -20°C overnight. To 100 μL of this solution CH_2_Cl_2_ (0,9 mL), 10% H_2_SO_4_ in methanol (2 mL) and the internal standards (100 μL of a 100 μg/mL solution of 14-methylhexadecanoic acid and 19-methylarichidic acid) were added. This reaction mixture was heated to 100°C for 1 hour. Then hexane (3 mL) and 5% NaCl (3 mL) was added to the samples. The hexane phase was dried and the samples were re-dissolved in hexane (500 μL) in order to separate and quantify the methylated fatty acids by gas chromatography-mass spectrometry (GC-MS). Three parallels where extracted from each sample before analysis. Standards of all fatty acids except 16:2, 16:3 and 18:4 were purchased from Sigma Aldrich (Billerica, MA, USA), 16:2, 16:3 and 18:4 were purchased from LGC Standards (Teddington, UK). The GC-MS analyses were performed on a Waters Quattro Premier GC (Waters, Milford, MA, USA) equipped with a 30 meters long fused silica Restek (Bellefonte, PA, USA) FameWax 0.25 mm column with 0.25 μm film thickness. The injector temperature was set to 250°C, the injection was in splitless mode and He 6.0 (Aga, Oslo, Norway) was used as carrier gas with a 1.0 mL/min constant flow. 1μL sample was injected, and the initial temperature on the column was 50°C. The initial temperature was held for 3 mins, then there was an increase of 2°C/min until the final temperature of 250°C was reached. The final temperature was held for 10 minutes and the total runtime was 113 mins. The GC-MS interface was kept at 250°C, and the mass spectrometer was equipped with an EI ionization source operated at 70 eV. The MS source temperature was 210°C and the trap current was 200 μA. The MS was run in full scan mode scanning m/z 150–400 with a scan time of 0.5 sec.

The quantification was based on relative peak area between the different analytes and the two internal standards. The choice of internal standard was based on retention time, and the FAs 10:0, 12:0, 14:0, 16:0, 16:1, 16:2, 16:3, 16:4, 18:0 and 18:1 were quantified with 14-methylhexadecanoic acid as internal standard, while the remaining longer chained FAs were quantified with 19-methylarichidic acid as internal standard. Quantification is based on the ratio of the area below the curve for the peak of the compound and the area below the curve for the peak of the respective internal standard. Standard curves were set up in the concentration range 10–1000 ng/mL, and all the standard curves had R^2^ values above 0.99 except 10:0 (0.987) and 18:4 (0.988). The quantification of 16:4 is semi-quantitative as it was not possible to find a commercial supplier of this FA. The quantification is based on the standard curve for 16:3 and gives an approximate concentration for 16:4, while the relative content of 16:4 between the different samples is correct. Each parallel sample was injected three times on the GC-MS, resulting in a total of nine injections from three parallels of each sample. Between every sample (nine injections) a blank sample was analysed to check for carry over effects, no carry over was observed. At regular intervals a standard solution of known concentration was injected to ensure the validity of the standard curves.

The method does not distinguish between the position of the double bonds in mono-, di-, tri- and tetraenes where there is more than one possible configuration, e.g. 18:1n-9 will not be separated from 18:1n-12.

All standards for the standard curve and the algae samples went through the same derivatization method to obtain FAMEs before analysis.

## Results and discussion

### Growth rate

From the bulk photosynthesis equation (H_2_O + CO_2_ + light → [CH_2_O]_n_ + O_2_), if CO_2_ was the limiting agent, additional supply would in theory increase the overall photosynthetic rate of algae [7]. Similarly, lowering of CO_2_ concentrations should lead to a decreased growth. The appropriate CO_2_ concentration seems to be a function of cell size, presence of extracellular carbonic anhydrase, intensity of CO_2_ supply and CO_2_ affinity level. Large cells, as a rule, have higher CO_2_ demands in order to satisfy their physiological needs at growth [22].

In the present study total lipid and fatty acids of two species representing different size ranges were investigated in terms of response to CO_2_ conditioning. The growth rate of the large species (*P*. *glacialis*) increased slightly after the 20–25% CO_2_ treatment, i.e. from 0.25 ± 0.09 to 0.29 ± 0.18 doublings day^-1^. However, the changes were not statistically significant (95% level, Students t-test). On the other hand, the small microalgae *A*. *longicornis* showed 50% decrease in its growth rate, i.e. from 0.58 to 0.25 ± 0.07 doublings day^-1^, but since only one replicate of the control growth rate is available, it is not possible to prove the statistical difference between the treatments. Wang et al. (2014) [23] demonstrated opposing results, indicating that a CO_2_ aeration of 10–20% boosted the growth of *Chaetoceros muelleri*. However, the authors also found that 30% CO_2_ was harmful for the alga, and reduced its growth rate if compared to the control. Chiu et al (2009) [3] demonstrated that a CO_2_ aeration level of only 5–15% was harmful for the green alga *Nannochloropsis oculata*, while Tang et al. (2007) [10] reported that *Chlorella pyrenoidosa* and *Scenedesmus obliquus* could grow well in CO_2_ levels as high as 30%. Thus, species –specific and, possibly, size—dependant response to different levels of CO_2_ supply was observed.

### Total lipid content

The results of the total lipid analysis showed that in cultures subjected to CO_2_ aeration the total lipid content increased (Table 2). *P*. *glacialis* showed a moderate increase in total lipid content from 8.91% (control) to 10.57% in cell dry mass in the CO_2_-aerated cultures. In *A*. *longicornis* the average lipid content increased even less, from 7.68% (control) to 8.23% However, only *P*. *glacialis* showed significant differences between the means (95% level, Student’s t-test). The reason of such moderate (> 2%) increase in the lipid content could be due to the fact that the fixed carbon is simultaneously channelled through the metabolic pathways of protein-, carbohydrate- and pigment synthesis [24]. However, the relative content of these supplementary compounds were not tested in the current study.

**Table 2:** Total lipid content and fatty acid composition of *A*. *longicornis* (Al) and *P*. *glacialis* (Pg) in controls (before CO_2_) and in CO_2_-treated cultures (after CO_2_). The percentage displays numbers as proportion of total lipids ± SD (n = 3).

Fatty acid	Al before CO2 (% TFA)	Al after CO2 (% TFA)	Pg before CO2 (% TFA)	Pg after CO2 (% TFA)
**C14:0**	8.78 ± 0.03	8.63 ± 0.28*	3.92 ± 0.36	4.17 ± 0.14
**C16:0**	8.54 ± 0.34	9.37 ± 0.30	12.04 ± 0.94	12.35 ± 0.66
**C16:1**	25.40 ± 0.25	23.96 ± 0.75*	16.79 ± 1.58	18.95 ± 0.47
**C16:2**	8.76 ± 0.11	7.87 ± 0.23*	5.15 ± 0.56	4.55 ± 0.17
**C16:3**	6.01 ± 0.09	5.68 ± 0.18*	8.52 ± 0.87	6.78 ± 0.10*
**C16:4**	3.78 ± 0.08	3.84 ± 0.15*	9.57 ± 1.02	10.03 ± 0.27
**C18:0**	3.48 ± 0.28	3.59 ± 0.77	2.65 ± 0.61	3.51 ± 0.69
**C18:1**	4.18 ± 0.15	3.90 ± 0.16*	1.69 ± 0.13	1.68 ± 0.04
**C18:2**	3.36 ± 0.12	2.47 ± 0.12*	0.25 ± 0.04	0.40 ± 0.02*
**C18:3**	2.63 ± 0.04	2.84 ± 0.05	4.76 ± 0.20	4.39 ± 0.42
**C18:4**	1.01 ± 0.05	0.80 ± 0.05*	4.17 ± 0.60	2.71 ± 0.07*
**C20:4**	0.07 ± 0.04	0.17 ± 0.01*	n. d.	n. d.
**C20:5 (EPA)**	19.09 ± 0.15	20.98 ± 1.19	26.59 ± 2.04	23.66 ± 0.38*
**C22:1**	0.13 ± 0.00	1.28 ± 0.64*	n. d.	n. d.
**C22:6 (DHA)**	4.04 ± 0.25	4.62 ± 0.18	3.90 ± 0.37	5.75 ± 0.30*
**C24: 1**	0.76 ± 0.20	n. d.	n. d.	0.93 ± 0.05
**Total saturates**	**20.71 ± 0.40**	**21.60 ± 1.00**	**18.63 ± 0.93**	**20.01 ± 0.79**
**Total monoens**	**30.66 ± 0.35**	29.14 ± 0.24*	**18.48 ± 0.50**	**21.58 ± 0.47**
**Total PUFAs**	**48.63 ± 0.32**	49.26 ± 1.17*	**62.89 ± 0.47**	**58.42 ± 0.39**
**Total lipid content (%)**	**7.68 ± 1.26**	**8.23 ± 0.49**	**8.91 ± 0.88**	**10.57 ± 0.59** *

* statistically significant difference (95% level, Student’s t-test)

Similarly, a study by Tang et al. (2011) [10] demonstrated that elevated CO_2_ levels (5–50%) enhanced total lipid accumulation in two microalgae*; Scenedesmus obliquus* and *Chlorella pyrenoidosa*. Though this increase was more pronounced (up to 9% wt.% at 50% CO_2_ aerated culture) if the maximum value is considered. Besides, the authors reported that CO_2_ aeration enhanced the accumulation of polyunsaturated fatty acids (PUFAs) in both species. Sydney et al. (2010) [25] found that the microalgae *B*. *braunii* and *D*. *tertiolecta* accumulated high amounts of lipids—33% and 11.44% accordingly, when cultivated at 5% CO2—enriched conditions. Yoo et al. (2010) [13] demonstrated that lipid productivity of *B*. *braunii* and *Scenedesmus* sp. increased significantly (1.9 fold and 3.7 fold accordingly) when cultivated in real flue gas containing approximately 5% CO_2_. Similar results were obtained by Nakanishi et al. (2014) [14] which showed an increase in lipid productivity up to 169.1 mg/L/d of *Chlamydomonas* sp. grown at 4% CO_2_. Chiu et al. (2007) [3] showed that 2% of CO_2_ led to maximum biomass and increased lipid productivity (0.142 g/L/d) in *Nannochloropsis oculata*.

### Fatty acid composition

As it can be seen from Table 2, the species demonstrated different patterns of response to CO_2_ aeration. Thus, in *A*. *longicornis* the content of EPA (20:5) increased from 19.09% to 20.98% (though statistically insignificant, p > 0.05), while the levels of palmitoleic acid (16:1) dropped from 25.40% to 23.96%. In *P*. *glacialis*, on the contrary, the production of EPA was reduced from 26.59% (in control) to 23.56% in the CO_2_-treated cultures, while the concentration of 16:1 insignificantly increased from 16.79% to 18.95%. This result is in coincidence with that obtained by Carvalho and Malcata (2005) [26] that have shown that the concentration of palmitoleic acid in the flagellate *Pavlova lutheri* increased with CO_2_-aeration, while EPA concentrations decreased. The concentration of another important omega-3 fatty acid, DHA (22:6), on the contrary, decreased with increasing CO_2_ concentrations. In our study the DHA profile changed differently in response to CO_2_ conditioning: while in *A*. *longicornis* no significant changes were observed between the controls and the treated cultures, in *P*. *glacialis* the concentration of DHA increased to almost the double of its initial value; from 3.90% to 5.86%.

The amount of 16:3 and 18:4 decreased (from 8.52% to 6.78% and from 4.17% to 2.71%, respectively) in CO_2_ treated cultures of *P*. *glacialis*. The same was the case for *A*. *longicornis*, though the changes in concentrations were minor. In contrast, the study by Wang et al. (2014) [23] demonstrated that the content of 16:3 and EPA in the diatom *Chaetoceros muelleri* showed positive correlation with the CO_2_ concentration, while the concentration of palmitic acid (16:0) demonstrated gradual decrease as the concentration of carbon dioxide raised.

In our study the concentration of 16:0 did not differ significantly between the controls and the CO_2_ treated cultures in either of the studied species (Table 1). Similarly, Tang et al. (2011) [10] demonstrated that the concentration of EPA increased with an increasing CO_2_ concentrations in cultures of *Scenedesmus obliquus*. Besides, the authors showed that the concentration of 16:3 nonlinearly increased as the CO_2_ concentration raised, while the concentration of 16:1, on the contrary, dropped (nonlinearly) with an increasing CO_2_ supply.

Our study demonstrated that total PUFAs concentration in CO2—aerated cultures of *A*. *longicornis* increased from 48.63% to 49.26%, while the concentration of monoens decreased slightly; from 30.66% to 29.14%. Such increase may be due to lowering of O_2_ concentration in the cultivation medium and as a consequence, increased activity of desaturation enzymes [10].

## Conclusions

The results of the current study demonstrated that cultures of *P*. *glacialis* had good tolerance to the CO_2_ levels as high as 20–25% in terms of growth rates. This means that CO_2_ mitigation can take place without any loss of lipid productivity. Moreover, CO_2_-conditioning helped to increase the total lipid content in this species. Though the EPA content possibly showed a slight decrease in the CO_2_-aerated culture, the concentration of another important omega-3 fatty acid, DHA, increased.

*A*. *longicornis* though was less tolerant to the tested CO_2_ levels, and seem to acts less robust in terms of growth sustainability.
